# The Evolution of Lateral Lumbar Interbody Fusion: A Journey from Past to Present

**DOI:** 10.3390/medicina60030378

**Published:** 2024-02-23

**Authors:** Anthony Xi Jie Wong, Derek Haowen Tang, Arun-Kumar Kaliya-Perumal, Jacob Yoong-Leong Oh

**Affiliations:** 1 Lee Kong Chian School of Medicine, Nanyang Technological University, Singapore 308232, Singapore; 2 Spine Division, Department of Orthopaedic Surgery, Tan Tock Seng Hospital, Singapore 308433, Singapore

**Keywords:** lumber interbody fusion, robotic surgical procedures, spinal navigation, spine, spondylosis

## Abstract

Lumbar interbody fusion procedures have seen a significant evolution over the years, with various approaches being developed to address spinal pathologies and instability, including posterior lumbar interbody fusion (PLIF), transforaminal lumbar interbody fusion (TLIF), anterior lumbar interbody fusion (ALIF), and lateral lumbar interbody fusion (LLIF). LLIF, a pivotal technique in the field, initially emerged as extreme/direct lateral interbody fusion (XLIF/DLIF) before the development of oblique lumbar interbody fusion (OLIF). To ensure comprehensive circumferential stability, LLIF procedures are often combined with posterior stabilization (PS) using pedicle screws. However, achieving this required repositioning of the patient during the surgical procedure. The advent of single-position surgery (SPS) has revolutionized the procedure by eliminating the need for patient repositioning. With SPS, LLIF along with PS can be performed either in the lateral or prone position, resulting in significantly reduced operative time. Ongoing research endeavors are dedicated to further enhancing LLIF procedures making them even safer and easier. Notably, the integration of robotic technology into SPS has emerged as a game-changer, simplifying surgical processes and positioning itself as a vital asset for the future of spinal fusion surgery. This literature review aims to provide a succinct summary of the evolutionary trajectory of lumbar interbody fusion techniques, with a specific emphasis on its recent advancements.

## 1. Evolution of Lumbar Interbody Fusion

Spinal fusion dates back to early 20th century, when Hibbs and Albee used fragments from the spinous process, laminae, and tibia as bone grafts to achieve posterior fusion of the spine, primarily in patients with tuberculosis [1,2]. Over time, fusion techniques evolved, and lumbar interbody fusion (LIF), which involves the insertion of a cage along with bone graft into the intervertebral space, became popular as a procedure offering both stability and fusion [3,4]. Early LIF procedures that were developed include PLIF by Cloward in 1943 [5], ALIF by Lane and Moore in 1948 [6], and TLIF by Harms and Rolinger in 1982 [7]. Brief descriptions of each of these procedures, as well as their advantages and disadvantages, are compiled in Table 1, as shown below. As highlighted, the LIF procedures are associated with certain advantages and disadvantages specific to each procedure. Posterior approaches, such as PLIF and TLIF, may affect posterior structures and the paraspinal musculature, and may cause retraction injury of the nerve roots and thecal sac [8,9,10]. While ALIF manages to avoid damaging the posterior structures, it may potentially damage intra-abdominal, intraperitoneal, and vascular structures [11,12,13,14,15]. Hence, there was a need for an alternative safer approach that reduces the risk of these complications.

## 2. A Safer Approach

### 2.1. The Extreme Lateral Interbody Fusion (XLIF) or Direct Lateral Interbody Fusion (DLIF)

Extreme lateral interbody fusion (XLIF), also known as direct lateral interbody fusion (DLIF), was developed by Pimenta in 2001 [22,23]. Instead of approaching the intervertebral disc anteriorly or posteriorly as in ALIF and PLIF, respectively, XLIF/DLIF accesses the intervertebral disc through a lateral retroperitoneal trans-psoas approach [24]. Ozgur et al., in 2006, further popularized the technique, especially the specialized retractors that can be utilized and the steps involved in the procedure [24,25], describing its suitability for accessing levels T12 to L5 [16]. While the indications for XLIF are mostly similar to those for any interbody fusion, such as spondylosis, spondylolisthesis, and degenerative scoliosis, there are certain contraindications to consider, including retroperitoneal scarring, abscesses, and abnormal vascular anatomy, such as aortic aneurysms [11,26].

### 2.2. The Procedure of XLIF/DLIF

The patient is generally placed in a right lateral decubitus position with the left side up. Strapping of the pelvis and chest wall is carried out to prevent changes in position, and adequate cushioning is provided at bony prominences. The operating table may be flexed to increase the distance between the iliac crest and rib cage. For a single-level exposure, a small incision is made on the lateral side over the affected disc space, utilizing X-ray guidance. A dilator is inserted through the incision, guided by the surgeon’s finger, to reach the psoas muscle while ensuring the protection of the peritoneum and abdominal contents. The psoas muscle is carefully parted between the middle and anterior third using blunt dissection, keeping the nerves posteriorly and great vessels anteriorly. The dilator is advanced through the psoas muscle, monitoring electromyography (EMG) responses to ensure safe passage protecting the lumbar plexus. Further dilators are inserted to gradually spread the psoas muscle until the retractor can be placed. After X-ray confirmation of retractor placement on the desired level, the retractor blades are expanded to adequately expose the disc space. Under direct vision, the disc is thoroughly removed, leaving the posterior annulus intact. A wide-bodied cage containing a bone graft is implanted supporting the lateral margins of the epiphyseal ring, restoring disc height and correcting imbalances [25,27,28,29]. Our procedure for XLIF is as originally described and can be found in the publication by Berjano et al. 2015 [30]. The procedure can be performed as a standalone technique or combined with lateral or posterior stabilization depending on the indication, the necessity for direct decompression, and the surgeon’s preference [31,32,33]. While X-ray guidance throughout the procedure is traditionally reliant on C-Arm or O-Arm, recent advancements have streamlined the integration of computer-assisted navigation, incorporating a navigated cobb, dilators, trails, and cage [34].

### 2.3. Benefits of XLIF over Other LIF Procedures

By utilizing the lateral retroperitoneal trans-psoas approach, XLIF avoids the risks of damaging the paraspinal muscles and the bony posterior elements as compared to PLIF and TLIF [35]. Unlike ALIF, XLIF does not require great vessel mobilization, and peritoneal structures are less likely to be injured [36]. In addition, preservation of the anterior and posterior longitudinal ligaments ensures stability of the treated levels [37]. Over the years, numerous studies have highlighted the effectiveness of XLIF in improving pain and disability scores, such as the Visual Analog Scale (VAS) and the Oswestry Disability Index (ODI), in addition to providing fusion and stability [29,37,38,39]. Studies conducted between 2015 and 2023, comparing XLIF with other LIF procedures, such as PLIF, TLIF, and ALIF, reveal several advantages of XLIF. These advantages include a lower risk of nerve damage, increased stability with greater segmental lordosis change, and reduced risks of subsidence. Additionally, XLIF has been associated with shorter hospital stays and lower estimated blood loss when compared to other LIF procedures. These findings are summarized in Table 2.

Cage subsidence is a common complication following LIF procedures, where the implanted cage sinks into the adjacent endplates, potentially compromising fusion [54]. If severe, it may also cause neural foraminal narrowing leading to nerve root compression, exacerbating pain and function [55,56]. While low bone mineral density and inappropriate cage positioning play crucial roles as risk factors in contributing to this problem, the intrinsic differences in cages used in PLIF or TLIF procedures also contribute significantly to a higher risk of cage subsidence compared to XLIF/OLIF cages [4,57]. 

Cages commonly used in TLIF and PLIF are the banana and bullet cages, respectively. These cages are smaller in size and, hence, the surface area that is in contact with the endplates is significantly less when compared to XLIF cages. There is also a reported increase in the risk of posterior cage migration when using smaller cages [58,59,60]. Given the smaller surface area in contact with the vertebral endplate, pressure dynamics lead to an increased force directly affecting the unsupported areas of the endplates, reducing overall stability [61]. Furthermore, the reduced stability could also be attributed to the resection of the posterior elements, especially the facet joints, ligamentum flavum, and posterior longitudinal ligament. 

On the other hand, while performing XLIF/DLIF, a wider (up to 26 mm) and longer (up to 60 mm) cage can be utilized, thereby improving endplate coverage and reducing subsidence risk [59,62,63]. This also allows sufficient distraction of the disc space and generates tension in the conserved ligaments, further enhancing stability. In a study conducted by Pimenta to elucidate the biomechanical stability of wide-bodied interbody cages inserted via lateral approaches compared to the smaller cages used in TLIF, it was demonstrated that the 26 mm wide bodied XLIF cage provided greater stability compared to the 11 mm wide TLIF cage [64]. In addition, our previous biomechanical analysis indicated that wide-bodied cages required three times the force of bullet cages for a 5 mm subsidence to occur, and constructs with wide bodied cages were 3.6 times stiffer than TLIF constructs [65].

### 2.4. Limitations of Trans-Psoas Approach

Despite its advantages, XLIF does also have its own limitations. Its main drawback is that it is commonly associated with postoperative hip flexion weakness (psoas weakness) due to the blunt dissection of the psoas muscle [66,67,68,69,70,71,72]. Most of these cases are transient and usually resolve within 2 weeks [73,74]. Despite neuromonitoring, the lumbar plexus may also be damaged, leading to lower limb weakness and paresthesia [75,76]. The lumbar plexus tends to adopt a more anterior location at lower spinal levels; hence, it is more prone to injury. Nevertheless, new research has shown that manipulating the entry site and psoas muscle traction direction may help reduce the risk of lumbar plexus injury [77]. Similarly, towards L4/L5 levels, the iliac vessels assume a more lateral course and, hence, may be damaged during XLIF surgery [78,79,80]. Moreover, XLIF is generally performed for L2–L5 levels, while it is contraindicated for the L5–S1 level. This is due to the risk of iliac vessels mobilization and the presence of the iliac crest restricting lateral access and interrupting cage insertion [81].

## 3. The Oblique Lumbar Interbody Fusion (OLIF)

**Figure 1:**
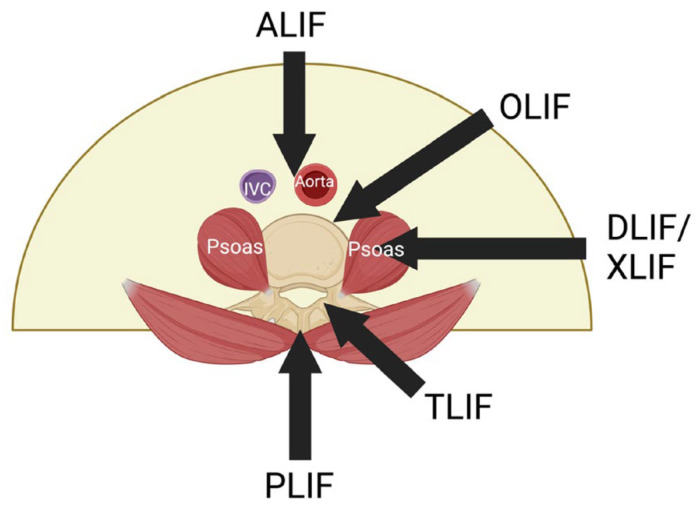
Graphic illustration of the OLIF approach in comparison to various other lumbar interbody fusion approaches. ALIF: anterior lumbar interbody fusion; OLIF: oblique lumbar interbody fusion; DLIF: direct lateral interbody fusion; XLIF: extreme lateral interbody fusion; TLIF: transforaminal lumbar interbody fusion; PLIF: posterior lumbar interbody fusion. Reproduced from Tan et al. 2023 [84].

Oblique lumbar interbody fusion (OLIF), also known as the anterior to psoas (ATP) approach for interbody fusion, is a procedure which was first adopted by Meyer in 1997, and the term was officially coined by Silvestre et al. in 2012. Subsequently, Hynes further developed and popularized the technique [82]. This approach typically involves minimally invasive access into the disc space via the anatomical corridor between the psoas muscle and the great vessels (aorta and inferior vena cava) and is suitable for performing fusion of levels L2–L5 [83]. In addition, Hynes also developed the concept of OLIF L5–S1, which is essentially an anterior approach performed in a lateral decubitus position when the L5–S1 region needs to be accessed [82]. A visual representation comparing the OLIF approach with various other lumbar interbody fusion techniques is provided in the graphic below (Figure 1).

### 3.1. The Procedure of OLIF L2-L5 [82]

**Figure 2:**
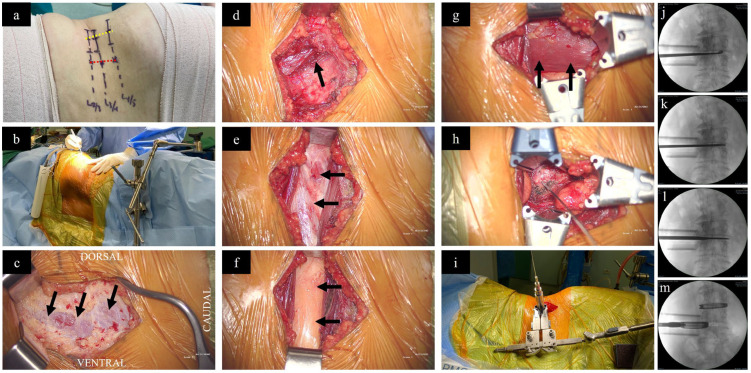
Surgical steps of OLIF. (**a**) While the patient is in a right lateral decubitus position, the surgical disc levels are marked (lines drawn across each disc level from anterior to posterior) under X-ray guidance. The red dotted line (3–5 centimeters anterior to the mid-disc) represents the incision site for OLIF, as performed for this patient, in relation to the yellow dotted line connecting the mid points of the disc levels, which represent the incision site for XLIF. (**b**) Surgeon standing on the abdominal aspect. (**c**) After incision of skin and subcutaneous tissue, the external oblique fascia is first encountered (arrows). (**d**) Following dissection of the external oblique, the internal oblique muscle is carefully split (arrow). (**e**) The transversalis fascia beneath the internal oblique muscle is exposed (arrows) (**f**) Blunt dissection of the transversalis fascia reveals the retroperitoneal fat (arrows). (**g**) By finger dissection, a plane is developed pushing the retroperitoneal fat anteriorly to reach the psoas muscle (arrows). (**h**) Placement of a guide wire into the disc space. (**i**) Application of the dilators and specialized retractor assembly. Preparation of the disc space using (**j**) curette, (**k**) disc punch, and (**l**) contralateral annular release using Cobb. (**m**) Placement of cages filled with bone graft under X-ray guidance.

For OLIF L2–L5, similar to XLIF, the patient should be positioned in a right lateral decubitus position on a radiolucent table to expose the spine from the left side, as the working passage between the psoas muscle and the IVC is narrower on the right side [85,86]. Once positioned, the legs are slightly flexed. A line is drawn across the desired disc level from the anterior to the posterior. This determines the incision, which is typically made approximately 3–5 cm anterior to the midpoint of the line (Figure 2a). The fascia of the external oblique muscle is first encountered and incised using electrocautery, followed by gentle finger dissection of the external oblique, the internal oblique, and the transversalis muscles (Figure 2c–e). While working on the transversalis fascia, the finger’s force is directed obliquely and posteriorly to prevent entry into the peritoneal cavity. Once the retroperitoneal fat plane is reached, the space should be developed both cephalad and caudal to the desired disc level (Figure 2f), followed by anterior retraction of the peritoneal sac and posterior retraction of the anterior belly of psoas muscle to establish the working corridor (Figure 2g). After retraction of the psoas muscle, the disc space is visualized and a guide wire is inserted, followed by a series of dilations to create space pushing aside the surrounding tissues (Figure 2h,i). Subsequently, a retractor is positioned over the dilators and can be anchored to the vertebral body using a pin. The retractor blades are oriented such that it allows for an orthogonal maneuver (rotating the instruments in a manner that they are obliquely inserted but become direct lateral as they go deeper) during disc removal, sequential trialling, and final placement of the interbody cage. Annulotomy and discectomy is performed under X-ray guidance (Figure 2j,k). After completing the disc preparation, a contralateral annular release is performed using a blunt-tipped shaver or cobb elevator, as carried out during XLIF (Figure 2l). Sequential trials distract the disc space and allow indirect decompression. Finally, a wide-bodied interbody cage is placed within the disc space (Figure 2m). The procedure may be accompanied by lateral or posterior stabilization, contingent upon the indication, the requirement for direct decompression, and the surgeon’s preference [87,88].

### 3.2. The Procedure of OLIF L5-S1 [82]

Surface marking is carried out with the help of X-ray guidance. A line is drawn across the L5-S1 disc level from the posterior to anterior and is extended onto the abdominal area. Subsequently, a second line is drawn from the center of the L5-S1 disc, projecting perpendicular to the floor onto the abdomen’s surface. Finally, approximately two finger-breadths anterior to the anterior superior iliac spine (ASIS), a third line is drawn connecting the first and second lines where the incision is made. The anatomical advantages of positioning the patient laterally enable abdominal contents to naturally fall away from the spine, resulting in a reduction in the need for peritoneal retraction. Dissection is performed as described for OLIF L2–L5. The common iliac artery pulse can be felt on the anterior border of the psoas, and the common iliac vein is medial to the artery. The adventitial layer containing the superior hypogastric plexus and sympathetic chain within is to be released by blunt dissection. After successfully releasing the adventitial layer, the left common iliac vein can be gently retracted laterally if needed. Basically, this approach accesses the L5-S1 disc in the classic supine ALIF interval below the bifurcation. However, here, a flexible semi-constrained retractor facilitates minimal tissue pressure and a minimally invasive approach. Discectomy and interbody cage placement are performed in a manner similar to ALIF.

### 3.3. Advantages of OLIF over XLIF

There are several factors that make OLIF more convenient compared to XLIF. Firstly, the surgical oblique approach enables direct and extensive visualization of crucial structures, such as the ureters, major blood vessels, and most of the psoas muscle, while XLIF provides only limited visualization [89]. It also allows for the visualization of the anterior disc margin, facilitating easier estimation of cage location and, hence, better anterior placement of cages [90]. The biggest advantage OLIF has over XLIF is that no dissection of the psoas is involved [91,92,93]. This facilitates limited EMG neuromonitoring of the psoas during the procedure [89,94]. However, some patients will still experience hip flexion weakness due to prolonged psoas retraction. Some nerve branches supplying the psoas traverse the intervertebral disc obliquely prior to ramification within the muscle and are, therefore, vulnerable to injury when muscle fibers of the superficial layer of psoas are pulled away from vertebral bodies [95,96,97]. Nonetheless, since most of the psoas muscle fibers are still preserved in OLIF, it is less associated with hip flexion weakness, with only up to 1.2–13.9% of patients experiencing postoperative hip flexion weakness as compared to 4.9–31.4% in XLIF [95,96,98,99,100,101,102,103,104]. As the lumbar plexus is also avoided, there is a reduced likelihood of lower limb sensory and motor weakness following OLIF as compared to XLIF. Various studies attribute the better postoperative VAS (Visual Analog Scale) and ODI (Oswestry Disability Index) scores following OLIF to the reduced incidence of psoas muscle injury [102].

### 3.4. Surgical Outcomes following OLIF

OLIF has been shown to be able to achieve similar surgical outcomes as compared to XLIF by the principle of indirect decompression [52,102,105,106]. There was no significant difference between the fusion rates of OLIF and XLIF [107]. OLIF achieved a similar restoration of disc height as XLIF, which has been determined to be the most significant factor in lumbar lordosis recovery. Some papers even suggested that OLIF leads to a greater increase in posterior disc space as compared to XLIF, along with reduced cage shift rates [102,108]. OLIF has also been shown to be effective in achieving greater sagittal deformity correction and lower risk of motor deficits compared to XLIF [109]. On its own, OLIF was demonstrated to be effective in elderly patients above 65 years old, in terms of clinical outcomes and patient satisfaction rates [110].

Similar to XLIF, OLIF is known to restore sagittal profiles and satisfies Schwab’s criteria for proper global alignment [111]. Proper global alignment has been linked to improved quality of life for adult patients with spinal deformities, especially spondylolisthesis [104,112,113,114,115,116,117]. Ko et al. measured the sagittal disc angle (SDA), coronal disc angle (CDA), mean disc height (MDH), and intervertebral foramen height (FH) directly after the XLIF and OLIF surgery, and one year postoperatively. OLIF had a significantly greater increase in sagittal disc angle on both occasions, while there were no significant differences between OLIF and XLIF for the other three parameters [118]. The table below highlights results from recent research comparing OLIF and XLIF (Table 3). 

### 3.5. Limitations of OLIF and Strategies to Overcome

Despite the use of wide interbody cages during OLIF and XLIF procedures, cage subsidence can still occur [124]. However, the incidence of subsidence is relatively lower than those observed after implanting smaller banana and bullet cages in other procedures, like PLIF and TLIF. Moreover, subsidence risk can be effectively reduced by taking into consideration several factors, such as a pre-existing bone health, conducting careful patient selection and evaluation of medical statuses, and practicing meticulous intraoperative techniques, such as avoiding aggressive endplate preparation [54,125]. The most frequently reported intraoperative complications are minor vascular injuries, mostly affecting segmental arteries, as well as endplate damage [126]. Other intraoperative complications, which occur in less than 1% of cases, include major vascular injury, vertebral body fracture, membrane laceration, and ureteral injury. The most common immediate postoperative complications are transient numbness or pain in the lower limbs, as well as temporary weakness and nerve deficits arising from sympathetic trunk injury [127,128]. However, a review demonstrated significant benefits via a modified OLIF procedure through the anteroinferior psoas approach that resulted in reduced psoas and neurovascular injury, as well as improved pain scores [129]. Only a small percentage of patients experienced ileus or infection. Other reported late postoperative complications included cage shifting, malposition or displacement, screw malposition or breakage, adjacent segment degeneration, and pseudarthrosis [130]. 

Given the above, multiple mitigating strategies have been employed to reduce these complications. For instance, reviewing of preoperative Magnetic Resonance Images (MRIs) and Computed Tomography (CT) scans taken in a right lateral decubitus position enables better appreciation of the lumbar vasculature and, thus, facilitates preoperative planning to minimize vascular damage [131]. The use of new design instruments has also greatly reduced approach-related complications, improving cage positioning as well as shortening operation times [132]. To avoid nerve damage, careful dissection of the anterior belly of psoas muscle restricted to the median coronal plane is performed to avoid injuries to the lumbar plexus [94]. Ureteric injuries may also be minimized by using a dual-phase contrast-enhanced CT scan and 3D reconstructed images to identify its anatomy, allowing for retraction of the retroperitoneal fatty tissue and anterior mobilization of the ureter prior to discectomy [133].

## 4. Recent Advances

### 4.1. Single-Position Surgery (SPS)—LLIF with Posterior Stabilization (PS)

Despite the significant demonstrated benefits of XLIF and OLIF, one limitation that both share is the need to reposition the patient when additional posterior decompression and stabilization needs to be performed [134]. The first stage of the surgery requires the patient to be placed in the lateral decubitus position to access the intervertebral space, discectomy, and cage placement. This is followed by the second stage, which requires the patient to be placed in a prone position for posterior decompression and stabilization using implants [135]. While doing so, re-draping and repositioning the patient prolongs surgical duration and may not be suitable for patients with contraindications [136,137]. Moreover, it is known that prolonged surgery increases the risk for surgical site infections (SSIs) [138,139]. To avoid this, some surgeons prefer to carry out the second stage of surgery on a different day. 

However, with the advent of single-position surgery (SPS), both XLIF and OLIF can be performed in a single position, predominantly the former, along with posterior stabilization (PS). This eliminates the need for patient repositioning, ultimately enhancing surgical efficiency and minimizing complications [135,140,141,142]. There are currently two main approaches to SPS: Lateral-SPS (L-SPS) and Prone–SPS (P-SPS), where the patient is placed either in the lateral decubitus or prone position, respectively, throughout the entire surgical duration. Both approaches are reported to have significant decreases in surgical times, with reductions from 60 min to up to 135 min, ultimately leading to a decrease in the duration of hospitalisation [136,142,143,144,145,146]. Furthermore, SPS has been demonstrated to reduce manpower necessary to perform the flip and importantly improves patient safety [137,147]. Regarding intraoperative blood loss, radiation exposure, complications, and reoperation rates, SPS offers similar or even better outcomes as that of dual-position surgery [145,148,149]. 

#### 4.1.1. Lateral Single-Position Surgery (L-SPS)

**Figure 3:**
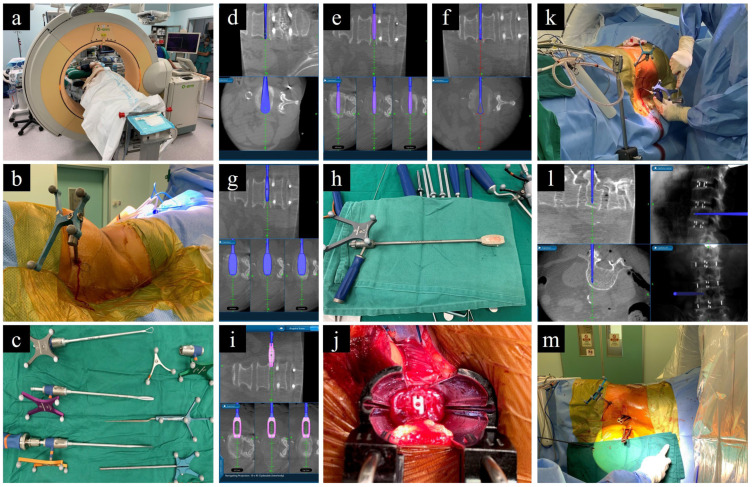
O-arm-based navigation-assisted L-SPS. (**a**) Patient positioning. (**b**) Reference frame on PSIS. (**c**) Navigated instruments for disc preparation. (**d**–**f**) Preparation of disc using cobb, shaver, and curette. (**g**) Trailing. (**h**,**i**) Navigated cage. (**j**) Intraoperative visualization of cage placement. (**k**–**m**) Application of pedicle screws under navigation guidance while patient is in lateral position.

In the context of L-SPS, the patient is consistently placed in a lateral decubitus position during the XLIF or OLIF procedures, which includes the application of pedicle screws for posterior stabilization (Figure 3). However, while this approach eliminates the need for flipping the patient before addressing the posterior pedicle screws, a common drawback arises. Surgeons may lack familiarity with performing posterior stabilization in the lateral position. Basic tasks, such as laminectomy for posterior decompression and the insertion of pedicle screws, become challenging, ultimately limiting the size of the posterior construct. Furthermore, there is limited lordosis correction, compared to that which can be accomplished in a prone position. These drawbacks have resulted in greater incidences of facet joint violation and pedicle screw breach [150]. Addressing these challenges requires a different approach to patient positioning that mitigates the shortcomings of lateral positioning while retaining the benefits of single-position surgery.

#### 4.1.2. Prone Single-Position Surgery (P-SPS) 

**Figure 4:**
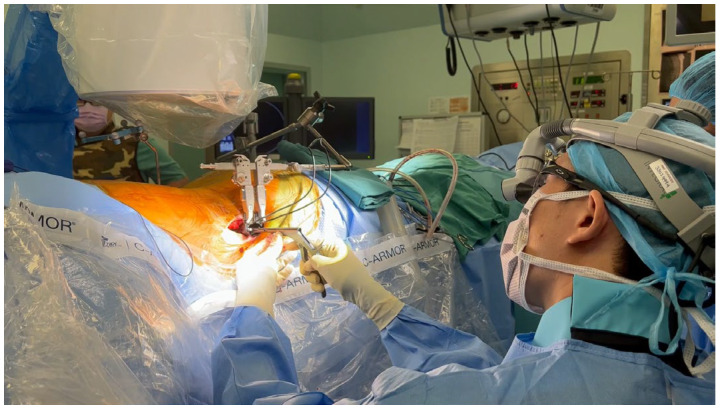
Prone single-position surgery (P-SPS), with the surgeon working on the lateral approach while the patient is in a prone position, facilitating the possibility of simultaneous posterior pedicle screw fixation.

P-SPS overcomes the aforementioned downsides of L-SPS, being procedurally similar to L-SPS, except that the patient is placed in a prone position instead of laterally (Figure 4). This positioning offers a more familiar and spacious area for the surgeon to operate, facilitating easier pedicle screw placement and posterior decompression, as deemed necessary [151,152,153]. Furthermore, studies have demonstrated that adopting a prone position enables enhanced correction of sagittal plane imbalance attributed to an augmented lumbar lordosis [148,154], resulting in better segmental lordosis correction when compared to L-SPS (Table 4) [155]. As the procedure evolved, accompanying advancements were made in the instruments designed to sustain a stable prone position amidst the new lateral forces [153]. In addition, specialized retractors were also developed to facilitate mid-disc docking and avoid posterior targeting [153]. However, it should be noted that cage placement in P-SPS is performed trans-psoas as in XLIF/DLIF. Here, plexus safety remains a concern. In order to overcome this, patient positioning with the hips in a neutral to extended alignment could lengthen the psoas muscle, causing it to shift more posteriorly along with the plexus [153]. Other limitations include surgeon ergonomics, challenges related to the depth of the surgical field in obese patients, absence of counter pressure during disc preparation and cage insertion, and difficulties in accessing the L4–L5 level due to the iliac crest [148,156]. Considering these limitations, P-SPS may not represent a comprehensive solution, but rather a viable and safe alternative that can be adopted based on patient characteristics, indications, and surgeon preferences. 

### 4.2. Robot-Assisted L-SPS and P-SPS

**Figure 5:**
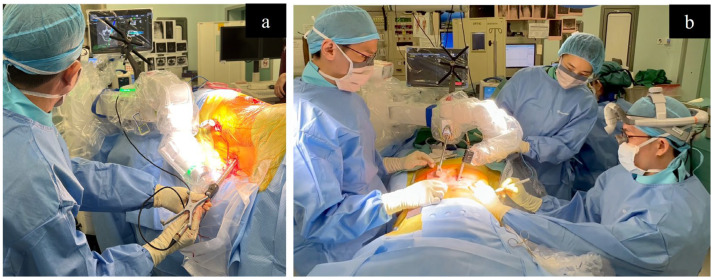
Simultaneous robotic single-position surgery (SR-SPS). (**a**) Robot-assisted application of pedicle screw while the patient is in a lateral position. (**b**) Robot-assisted application of pedicle screws while a second surgeon simultaneously performs a lateral approach with the patient in a prone position.

The use of robots in spine surgery is gaining popularity, evolving from the era of computer-assisted navigation. With the adoption of a preoperative planning software and robotic guidance for pedicle screw placement, there is an enhanced ability to adhere to and execute the surgical plan with the utmost accuracy [161]. This improves the likelihood of success and reduces the potential for significant complications. Studies have also emphasized the potential for decreased blood loss and shorter perioperative hospital stays achievable through the use of robots [162]. In our institute we use the Mazor X Stealth Edition Robot (Medtronic). In short, following preoperative planning, the procedure begins with establishing a stable bed and securing the patient for robotic precision throughout the surgery. Subsequently, the robotic arm performs a 3D mapping of the operative field, and the patient is registered with the O-arm or fluoroscopy, independently registering each vertebra and correlating them with the previously obtained CT scan. The reference frame is secured in place, and snapshot tracker registration is performed. For screw application, the robotic arm moves along the preplanned trajectory with precision, enabling the use of instrumentation through the arm. Navigated instruments are then introduced through the robotic arm into the pedicle, preparing it for screw application, with the robotic arm maintaining a fixed trajectory [163]. These robots are being utilized both during L-SPS and P-SPS (Figure 5) [164]. With two surgeons, it becomes feasible to simultaneously perform robotic posterior pedicle screw application and lateral cage placement with the patient in a single position (simultaneous robotic single-position surgery; SR-SPS).

Huntsman et al. reported the early results of a series of 55 cases wherein L-SPS was performed by a single surgeon starting with pedicle screw placement under robotic guidance, followed by LLIF, and, finally, fixation of rods to complete the construct [165]. Of the 328 screws inserted, 2% were repositioned at the surgeon’s discretion, resulting in a success rate of 98% for navigated robot-assisted pedicle screw placement. Diaz-Aguilar et al. published a series of cases in which two surgeons conducted SR-SPS while the patient is in lateral position. This approach effectively reduced the overall operating time, with one surgeon concentrating on the LLIF procedure, while the other focuses on the robotic screw placement [164]. During such procedures, utmost care needs to be taken not to shift or move the patient during the procedure to avoid introducing mismatch error affecting the robotic guidance [164]. These studies, in addition to several others, underscore the safety and clinical effectiveness of SPS utilizing technological advancements [163,166,167]. As the technique continually evolves, the future may bring forth additional advancements, making it safer and more accessible. Despite limited studies investigating the financial impact of incorporating robotic technology into these procedures, available evidence indicates its cost-effectiveness [168]. With ongoing developments and the increased availability of robotic technology, this procedure has the potential to establish itself as the standard in the future.

## 5. Conclusions

Lumbar interbody fusion has undergone significant transformation over the years and remains a dynamically advancing field. The advent of LLIF has led to a significant reduction in the associated risks compared to other LIF techniques, like ALIF, PLIF, and TLIF. Among the LLIF techniques, both XLIF/DLIF and OLIF are currently being utilized, at the surgeon’s discretion, with OLIF gaining increasing recognition for its advantages over XLIF/DLIF. Single-position LLIF with PS has eliminated the necessity for patient repositioning and has substantially reduced operative durations. Ultimately, given the wide armamentaria of lumbar interbody fusion techniques and their numerous associated modifications available from inception until the present day, the choice of approach depends importantly on patient factors and safety, as well as the surgeon’s preference and relative expertise in performing the procedure. To that end, ongoing research is focused on improving LLIF procedures by minimizing related complications, and the recent utilization of robots has greatly streamlined these processes, positioning them as a valuable asset for the future. Although this review is limited by a lack of quantitative synthesis, it offers a narrative exploration of the evolutionary trajectory of LLIF over time, providing qualitative insights into the research area.

## Figures and Tables

**Table 1:** Lumbar interbody fusion techniques: PLIF, TLIF, and ALIF [16,17,18,19,20,21].

	Procedure Description	Advantages	Disadvantages
PLIF	Posterior midline incision in prone position; requires laminectomy and retraction of thecal sac and nerve roots to reach the intervertebral disc space	▪ Favors adequate visualization of the thecal sac and nerve roots ▪ Allows direct decompression of the spinal canal and nerve roots	▪ Risk of damage to thecal sac and nerve roots during retraction ▪ Paraspinal scarring ▪ Limited coronal correction ▪ Allows insertion of only smaller cages
TLIF	Posterior incision with a more lateral trajectory; requires facetectomy to allow visualization of nerve roots and perform discectomy	▪ Limited retraction of nerve roots ▪ Preservation of posterior midline structures ▪ Can be performed as a minimally invasive procedure	
ALIF	Longitudinal midline or paramedian incision to access retroperitoneal space in supine position	▪ Spares paraspinal musculature ▪ Preservation of posterior elements ▪ Allows for direct implantation of a wide-bodied cage ▪ Optimal restoration of lordosis	▪ High risk of injury to visceral and vascular structures due to mobilization of great vessels ▪ Sympathetic hypogastric plexus injury

**Table 2:** Studies analyzing XLIF versus other LIF procedures (PLIF, TLIF, and ALIF).

Study	Patients	Results	Conclusions
Saadeh et al. (2019) [40]	40 (20 XLIF, 20 TLIF)	**Segmental lordosis change:** XLIF: 4.9° TLIF: 2.6°	XLIF achieved greater segmental lordosis correction as compared to TLIF
Jain et al. (2018) [41]	33 (17 XLIF, 16 TLIF)	**Blood loss**: XLIF: 36 ± 16 mL TLIF: 700 ± 767 mL **Length of hospital stay**: XLIF: 2.6 ± 2.9 days TLIF: 3.3 ± 0.9 days	The XLIF group experienced decreased blood loss and shorter hospital stays compared to the TLIF group
Xu, Bach, and Uribe. (2018) [42]	24 (16 XLIF, 8 ALIF)	**Blood loss:** XLIF: 60.6 mL ALIF: 106.3 mL	The XLIF group experienced decreased blood loss compared to the ALIF group
Ye, Hu, Zhang, and Xu. (2019) [43]	2625 (1081 XLIF, 241 ALIF, 1303 PLIF/TLIF)	**Length of hospital stay:** XLIF: 3.77 days PLIF/TLIF: 4.04 days ALIF: 4.31 days	The XLIF group had a shorter hospital stay in comparison to the PLIF/TLIF and ALIF groups
Hrabek et al. (2015) [44]	431 (101 XLIF, 330 ALIF)	**Sympathectomy risk percentages:** XLIF: 4% ALIF: 15%	XLIF has a lower sympathectomy risk than ALIF
Sembrano et al. (2016) [45]	55 (29 MIS XLIF, 26 MIS TLIF)	**Surgical duration:** MIS XLIF: 171 min MIS TLIF: 186 min **Blood loss < 100 mL:** MIS XLIF: 79% MIS TLIF: 27%	The XLIF group had notably less blood loss in comparison to the MIS TLIF group
Isaacs, Sembrano, Tohmeh, and Group. (2016) [46]	55 (29 XLIF, 26 MIS TLIF)	**Average disc height change (24-month assessment):** XLIF: −0.9 MIS TLIF: −1.7 **Graft subsidence percentage (24-month assessment):** XLIF 3% MIS TLIF 10%	XLIF demonstrates reduced subsidence and less resultant disc height loss compared to the MIS TLIF group at 24 months post-surgery
Lu and Lu. (2019) [47]	Finite element analysis (computerized models)	**Stress peaks in endplate and cancellous bone, respectively:** TLIF: 24.94–60.03 MPa, 0.72–1.96 MPa; XLIF: 17.01–35.32 MPa, 0.56–1.12 MPa	XLIF exhibits fewer stress peaks in the cortical endplate and cancellous bone in comparison to TLIF, which helps lower the risk of subsidence while preserving disc height and segmental angle
Zhang, Bai, Dokos, Cheung, and Diwan. (2019) [48]	Finite element analysis (computerized models)	**Maximum stress and strain** presented at the rods and facet joint, respectively, while utilizing an XLIF cage was less compared to when utilizing a TLIF cage	XLIF induces lower stress and strain on the fixed segments compared to TLIF and, hence, achieves greater stability
Ohba, Ebata, and Haro. (2017) [49]	102 (46 XLIF, 56 PLIF)	**Blood loss:** XLIF: 51 ± 41 mL PLIF: 206 ± 191 mL	The XLIF group had notably less intraoperative blood loss in comparison to the PLIF group
Goodnough et al. (2019) [50]	75 (21 XLIF, 54 ALIF)	**Blood loss:** XLIF: 50–100 mL ALIF: 150–400 mL	The XLIF group had notably less intraoperative blood loss in comparison to the ALIF group
Sembrano, Yson, Horazdovsky, Santos, and Polly, Jr. (2015) [51]	85 (35 XLIF, 50 TLIF)	**Mean operative level segmental lordosis:** XLIF: 3.2 ± 3.6° TLIF: 1.9 ± 3.9° **Overall lumbar lordosis change:** XLIF: 2.5 ± 4.1° TLIF: 2.1 ± 6.0°	XLIF provided better segmental lordosis in comparison to TLIF
Yingsakmongkol et al. (2022) [52]	60 (30 XLIF, 30 MIS TLIF)	**Estimated blood loss:** MIS TLIF: 200.33 ± 59.22 mL XLIF: 49.17 ± 32.91 mL **Duration of hospital stay:** MIS TLIF: 4.33 ± 0.61 days XLIF: 3.6 ± 0.62 days **Operative time:** MIS TLIF: 2.82 ± 0.47 h XLIF: 2.4 ± 0.81 h	The XLIF group exhibited decreased blood loss, as well as shorter postoperative hospital stays and operating times, when compared to TLIF
Kono, Gen, Sakuma, and Koshika. (2018) [53]	40 (20 XLIF, 20 TLIF)	**Estimated blood loss:** XLIF: 36.1 ± 15.3 mL TLIF: 225.7 ± 215.9 mL **Change in disc height (12-month assessment in comparison to pre-op status):** XLIF: 1.8 ± 1.9 mm TLIF: 0.7 ± 1.4 mm	The XLIF group had significantly lower blood loss compared to the TLIF group, and the post-procedure disc height remained well-maintained at the 12-month assessment

**Table 3:** Recent research on the comparison of OLIF and XLIF.

Study	Patients	Results	Conclusions
Li, Zhang, and Shen. (2019) [102]	2605 (1043 OLIF, 1562 XLIF)	**Transient psoas weakness:** OLIF: 8.8% XLIF: 21.2%	The OLIF group experienced less transient psoas weakness than the XLIF group
Walker et al. (2019) [103]	6481 (1874 OLIF, 4607 XLIF)	**Transient psoas weakness:** OLIF: 5.7% XLIF: 19.7%	
Emami et al. (2023) [119]	1010 (408 OLIF, 602 XLIF)	**Rate of neuropraxia:** OLIF: 10.9% XLIF: 21.2%	OLIF group experienced a lower rate of neurological complications than the XLIF group
Kim, Chang, Chang. (2022) [120]	287 OLIF 584 XLIF	**Fusion rates:** OLIF: 96.9% XLIF: 91.6%	Similar fusion rates between OLIF and XLIF
Ko, Park, Kim. (2019) [118]	343 (142 OLIF, 201 XLIF)	**OLIF group:** SDA: 11.1 ± 3.4 CDA: 0.9 ± 1.2 MDH: 12.2 ± 1.5 FH: 20.7 ± 2.0 **XLIF group:** SDA: 8.4 ± 3.5 CDA: 0.9 ± 1.2 MDH: 12.0 ± 1.6 FH: 21.7 ± 2.4	OLIF has similar CDA, MDH, and FH but higher SDA as compared to XLIF
Yingsakmongkol et al. (2022) [52]	60 patients (30 OLIF, 30 XLIF)	**Estimated blood loss:** OLIF: 48.67 ± 33.4 mL XLIF: 49.17 ± 32.91 mL **Difference in clinical pain parameters (1-year assessment)** OLIF: VAS back: −7.47 (−8.08 to −6.85) VAS leg: −7.67 (−8.33 to −7.01) ODI: −52.76 (−59.66 to −45.85) XLIF: VAS back: −8.3 (−8.86 to −7.74) VAS leg: −7.23 (−7.9 to −6.57) ODI: −49.65 (−57.64 to −41.67)	OLIF showed comparable blood loss to that of XLIF, and there is no significant contrast in postoperative clinical pain parameters between the two procedures at the 1-year assessment
Jin et al. (2018) [121]	43 patients (22 MIS XLIF, 21 MIS OLIF)	**Persistent postoperative complications:** MIS XLIF: 13.6% MIS OLIF: NIL	MIS OLIF results in fewer long-lasting postoperative complications compared to MIS XLIF
Miscusi et al. (2018) [122]	45 patients (31 XLIF, 14 OLIF)	**Post-op ODI scores:** OLIF: Better (71.4%); Stable (21.5%) XLIF: Better (22.6%); Stable (61.2%) SF-36 Mental scale (at follow-up): OLIF: 70.00% XLIF: 60.22% SF-36 Physical scale (at follow-up): OLIF: 55.00% XLIF: 51.15%	The OLIF group demonstrates superior ODI and SF-36 scores when compared to the XLIF group
Fujibayashi et al. (2017) [98]	2998 patients (1995 XLIF, 1003 OLIF)	**Complication rates:** OLIF: 15.3% XLIF: 19.4% **Psoas weakness:** OLIF: 3.0% XLIF: 4.9% **Neuromonitoring usage:** OLIF: 18.8% XLIF: 99.3%	The OLIF group exhibits a reduced overall complication rate in comparison to the XLIF group The incidence of psoas weakness is lower in the OLIF group than in the XLIF group OLIF necessitates limited neuromonitoring compared to XLIF
Ricciardi et al. (2023) [123]	318 patients (128 OLIF, 190 XLIF)	**Psoas weakness:** OLIF: 1.56% XLIF: 7.37% **Neurological symptoms:** OLIF: 3.9% XLIF: 13.1%	The OLIF group had fewer patients with psoas weakness and neurological symptoms compared to the XLIF group
Aleinik et al. (2021) [104]	Review of 2900 patients (17 sources)	**Overall complication rate** following OLIF is 13.9% **Incidence of severe persistent complications** following OLIF is less than 1%	The relatively low complication rate makes OLIF superior to other approaches

**Table 4:** Recent research on prone single-position surgery (P-SPS).

Study	Patients	Results	Conclusions
Lamartina et al. (2020) [157]	17 patients; 7 P-SPS and 10 conventional XLIF with PS	Oswestry Disability Index (preoperative to postoperative): P-SPS: 48.5 ± 21 to 14.57 ± 18.54 Conventional XLIF: 50.8 ± 11.7 to 22.50 ± 13.9 Back Pain Numeric Rating Scale (preoperative to postoperative): P-SPS: 7.7 ± 1.7 to 1.71 ± 2.91 Conventional XLIF: 5.7 ± 1.2 to 3.7 ± 2.91 Leg Pain Numeric Rating Scale (preoperative to postoperative): P-SPS: 8.5 ± 1.2 to 2.71 ± 3.25 Conventional XLIF: 7.2 ± 1.3 to 2.50 ± 3.03	P-SPS is feasible and safe; results are comparable to the standard technique
Pimenta et al. (2021) [158]	32 patients; 45 levels	Index level segmental lordosis increased from 8.7° pre-operatively to 14.8° postoperatively; lumbar lordosis (L1-S1) increased from 41.9° pre-operatively to 46.7° postoperatively; preoperatively, 22 patients had a pelvic incidence (PI)–lumber lordosis (LL) mismatch of 10° or more, while postoperatively, only 12 patients had a mismatch beyond 10°	P-SPS is associated with a significant gain of segmental lordosis and correction of spinopelvic alignment parameters
Pimenta et al. (2021) [152]	27 patients (L4-5 only: 18 patients; L3-5: 8 patients and L2-5: 1 patient)	Posterior stabilization: 22 patients (81.5%) Mean surgical duration: 182 ± 72 min Mean trans-psoas time: 29 ± 14 min Estimated blood loss: 200 ± 166 mL Median hospitalization time: 2 days Onset of sensory deficit: 2 patients Onset of a motor and sensory deficit: 1 patient	P-SPS is safe and feasible for approaching the L4-5 disk, presenting with low rate of complications and new-onset neurologic deficits
Smith et al. (2021) [153]	120 patients; 176 levels; 22 surgeons	Lateral exposure: 18 min/level Retraction time: 25 min/level Percutaneous pedicle screws: 65% Open pedicle screws: 24% Direct decompression: 37% Osteotomy or bony releases: 9%	Perioperative outcomes of P-SPS are consistent with lateral decubitus experience
Soliman et al. (2022) [159]	22 patients; 11 P-SPS and 11 TLIF	Improvement in LL: P-SPS: 11.5 ± 9.5 TLIF: 0.1 ± 15.1 Postoperative PI-LL: P-SPS: 3 ± 10.3 TLIF: 14.9 ± 14.1 Change in PI-LL: P-SPS: 15.5 ± 7.7 TLIF: 3.8 ± 15.2	P-SPS led to superior enhancements in both postoperative radiographic parameters and patient-reported outcomes
Soliman et al. (2022) [160]	20 patients; 10 P-SPS and 10 conventional XLIF with PS	Improvement in LL: P-SPS: 9.9 ± 8.5 Conventional XLIF: 0.5 ± 11	P-SPS group demonstrated a significantly better improvement in lumbar lordosis
Amaral et al. (2023) [155]	71 patients; 18 P-SPS and 53 conventional XLIF with PS	After propensity score matching: P-SPS (n = 18): 6.6° ± 5.5° Conventional XLIF (n = 18): 1.9° ± 4.7°	P-SPS can significantly enhance segmental lordosis correction

## Data Availability

Data sharing is not applicable to this article.
